# Comparative Chloroplast Genome Analyses of Species in *Gentiana* section *Cruciata* (Gentianaceae) and the Development of Authentication Markers

**DOI:** 10.3390/ijms19071962

**Published:** 2018-07-05

**Authors:** Tao Zhou, Jian Wang, Yun Jia, Wenli Li, Fusheng Xu, Xumei Wang

**Affiliations:** 1 School of Pharmacy, Xi’an Jiaotong University, Xi’an 710061, China; zhoutao196@mail.xjtu.edu.cn (T.Z.); wangjian6318@126.com (J.W.); lwl3659003@stu.xjtu.edu.cn (W.L.); xfs19940903@stu.xjtu.edu.cn (F.X.); 2 Key Laboratory of Resource Biology and Biotechnology in Western China (Ministry of Education), School of Life Sciences, Northwest University, Xi’an 710069, China; jy878683@163.com

**Keywords:** *Gentiana* section *Cruciata*, chloroplast genome, molecular markers, species authentication

## Abstract

*Gentiana* section *Cruciata* is widely distributed across Eurasia at high altitudes, and some species in this section are used as traditional Chinese medicine. Accurate identification of these species is important for their utilization and conservation. Due to similar morphological and chemical characteristics, correct discrimination of these species still remains problematic. Here, we sequenced three complete chloroplast (cp) genomes (*G. dahurica*, *G. siphonantha* and *G. officinalis*). We further compared them with the previously published plastomes from sect. *Cruciata* and developed highly polymorphic molecular markers for species authentication. The eight cp genomes shared the highly conserved structure and contained 112 unique genes arranged in the same order, including 78 protein-coding genes, 30 tRNAs, and 4 rRNAs. We analyzed the repeats and nucleotide substitutions in these plastomes and detected several highly variable regions. We found that four genes (*accD*, *clpP*, *matK* and *ycf1*) were subject to positive selection, and sixteen InDel-variable loci with high discriminatory powers were selected as candidate barcodes. Our phylogenetic analyses based on plastomes further confirmed the monophyly of sect. *Cruciata* and primarily elucidated the phylogeny of Gentianales. This study indicated that cp genomes can provide more integrated information for better elucidating the phylogenetic pattern and improving discriminatory power during species authentication.

## 1. Introduction

*Gentiana* is the largest genus in the family Gentianaceae and widely distributed throughout the northern Hemisphere [1]. Approximately 362 species are recognized in genus *Gentiana* which have been divided into 15 sections [2]. Section *Cruciata* contains 21 species which are mainly distributed in eastern Eurasia [3]. Most species of this section are restricted to alpine regions, although some of them could be found at altitudes below 1000 m at higher latitudes [1]. Four species (*G. macrophylla*, *G. crassicaulis*, *G. straminea*, and *G. dahurica*) in sect. *Cruciata* are used as the original plants of traditional Chinese medicine named Qin-jiao [4]. The roots of these plants contain abundant secoiridoid active compounds which could be used for the treatment of diabetes, apoplexy, paralysis, and rheumatism [5,6,7,8].

Recently, the wild resources of some *Gentiana* species are dramatically declined due to overexploitation and some of them have been listed in the National Key Protected Wild Herbs in China [5,7]. However, the demand of natural sources for these plants remains high due to the high pharmacological and economical values. Therefore, many economically motivated adulterants of Qin-jiao products with similar morphological characters have been developed to substitute the genuine medicinal materials. Generally, the authentication of herbs was based on the morphological and histological inspection. But these methods may not be suitable for authenticating some species in sect. *Cruciata* due to the following reasons. Firstly, most species of sect. *Cruciata* shared the similar morphological characters especially in terms of leaf shape. Secondly, some species in this section are usually located in the sympatric distributions, thus intermediate morphology could be detected due to interspecific hybridization [9,10]. Thirdly, pharmacognostical studies showed that some species such as *G. siphonantha* and *G. straminea* usually shared similar chemical profiles [11]. Some other factors, such as growth conditions, developmental stage, and internal metabolism may affect the secondary metabolite accumulation in Qin-jiao and limit the application of such chemical analyses for authenticating the species of sect. *Cruciata*. In addition, chemical methods for identifying the medicinal plants are also expensive and not suitable for high-throughput analysis [12]. Therefore, reliable and cost-efficient methods are needed to authenticate the medical plants of sect. *Cruciata*.

Chloroplast (cp) genome of angiosperm is characterized by a typical quadripartite structure that contains a pair of inverted repeat (IR) regions separated by a large single-copy (LSC) and a small single-copy (SSC) region [13], and it is highly conserved compared to nuclear and mitochondrial genomes. Although chloroplast genomes are highly conserved, some hotspot regions with single nucleotide polymorphisms and insertion/deletions could be found and these regions may provide enough information for species identification [14,15]. Due to low recombination, uniparental inheritance, and low nucleotide substitution rates, many cp genetic markers have been used for plant phylogenetic, phylogeographic, and population genetic analyses [16]. It has been proven that some chloroplast sequences such as *trnH-psbA*, *rbcL*, and *matK* were commonly used as DNA barcodes for plants discrimination [17]. But in some cases, above commonly used DNA barcodes were not suitable to distinguish closely related plants due to limited variation loci [16,18]. Recently, it has been proposed that the complete cp genome could be used as a plant barcode, and various research have demonstrated that complete cp genome can greatly increase resolution for resolving difficult phylogenetic relationships at lower taxonomic levels [16,19,20,21,22]. In addition, using the cp genome as a genetic marker for identifying the plant will avoid the problems such as gene deletion and low Polymerase Chain Reaction (PCR) efficiency [23].

Most species in section *Cruciata* were recently diverged and originated from a common radiation in the Qinghai-Tibet Plateau (QTP) before the Pleistocene [1,10], therefore these species were usually closely related and showed parallel evolutionary relationships [1]. Previous research showed that commonly used DNA barcodes in some cases may not be suitable to identify the medicinal plant of this section [24,25]. Therefore, more specific barcodes with enough variation are needed to discriminate closely related species belong to sect. *Cruciata*. Nowadays, with the improvement of sequencing and assembly technologies, it is comparatively simple to obtain comprehensive chloroplast sequences for identifying *Gentiana* species. By utilizing the variable information provided from cp genomes, we can not only obtain more specific barcodes for species authentication in sect. *Cruciata*, but also shed light on the complex evolutionary relationships of the species in this section.

In the present study, we obtained the chloroplast genome sequences of *G. dahurica*, *G. siphonantha* and *G. officinalis* by using de novo assembly of whole-genome sequencing (WGS) data derived from high throughput sequencing technology. We also comparatively analyzed the chloroplast genomes of eight species in sect. *Cruciata* and developed credible cp genome derived InDel markers to authenticate these species. These markers are not only valuable tools for further evolutionary and population genetic studies on *Gentiana*, but also could be used as standardized barcodes for authenticating the original plants of Qin-jiao.

## 2. Results

### 2.1. Complete Chloroplast Genome Features of Sect. Cruciata

**Figure 1:**
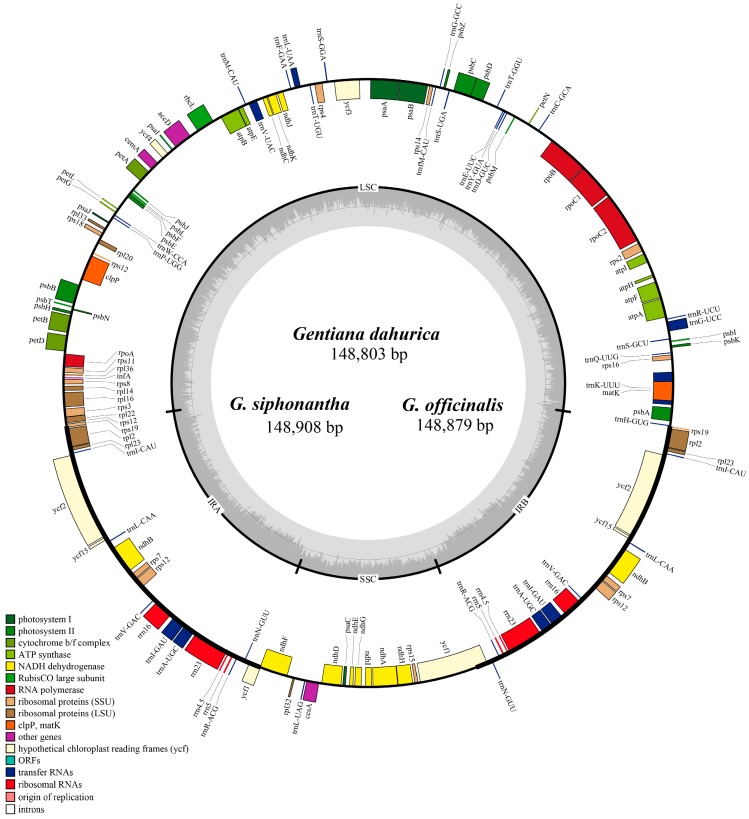
Merged gene map of the complete chloroplast genomes of three *Gentiana* species. Genes belonging to different functional groups are classified by different colors. The genes drawn outside of the circle are transcribed counterclockwise, while those inside are clockwise. Dashed area in the inner circle represent GC content of chloroplast genome.

**Figure 2:**
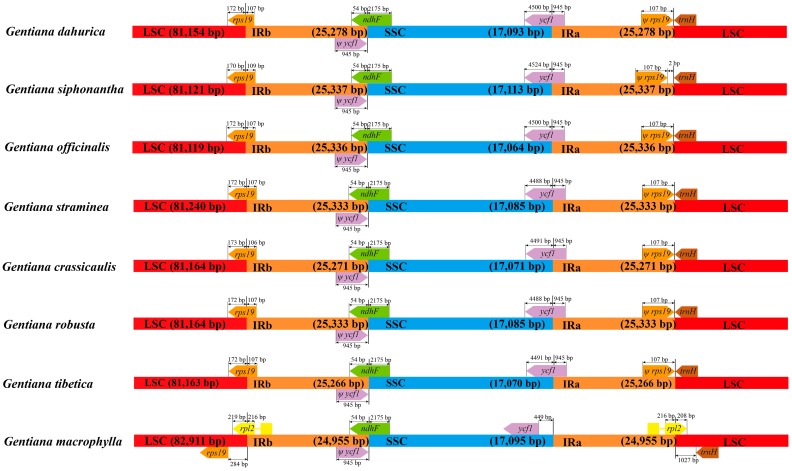
Comparison of chloroplast genome borders of LSC, SSC, and IRs among eight species in *Gentiana* sect. *Cruciata*. *Ψ* indicates a pseudogene.

The chloroplast genomes of *G. dahurica*, *G. siphonantha*, and *G. officinalis* were sequenced with approximately 5.2, 5.8, and 5.6 Gb of paired-end reads, respectively. The raw reads with a sequence length of 125 bp were trimmed to generate the clean reads for the next assembly. After quality filtering, 10,114,902, 11,405,694, and 11,288,676 clean reads were recovered for *G. dahurica*, *G. siphonantha* and *G. officinalis*, respectively. Combined with the de novo and reference guided assembly, the cp genomes were obtained. The four junction regions between the IRs and SSC/LSC regions were confirmed by PCR amplification and Sanger sequencing. We mapped the obtained sequences to the new assembled genomes and no mismatch or InDel was observed. We compared the basic genome features of three newly sequenced cp genomes with five previously published cp genomes [26,27,28] and found that all the chloroplast genomes possessed the typical quadripartite structure with the length range from 148,765 to 149,916 bp (Table 1, Figure 1). The whole cp genome contained a pair of inverted repeat regions (IRs: 24,955–25,337 bp) which were separated by a small single copy region (SSC: 17,070–17,095 bp) and a large single copy region (LSC: 81,119–82,911 bp) (Table 1). Although genomic structure and size were highly conserved in eight cp genomes, the IR/SC boundary regions still varied slightly (Figure 2). All the eight chloroplast genomes contained 112 unique genes arranged in the same order, including 78 protein-coding genes, 30 tRNA genes, and 4 rRNA genes. Two genes (*rps16*, *infA*) were inferred to be pseudogenes (Figure S1). The overall guanine and cytosine (GC) content in each chloroplast genome is identically 37.7% (Table 1).

### 2.2. Comparative Analyses of the Chloroplast Genomes of Species of Sect. Cruciata

**Figure 3:**
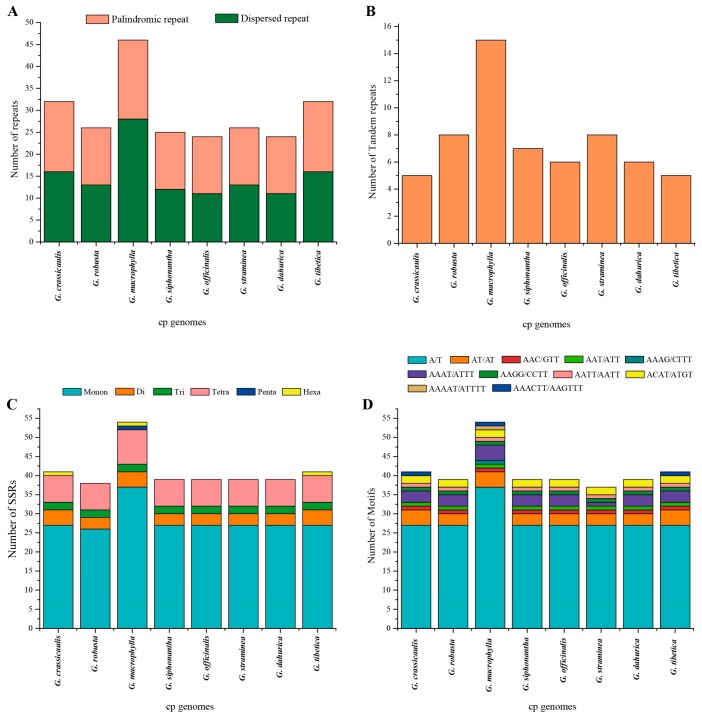
Analysis of different repeats in eight chloroplast genomes of *Gentiana* sect. *Cruciata*. (**A**) Histogram showing the number of palindromic repeats and dispersed repeats; (**B**) histogram showing the number of tandem repeats; (**C**) number of different simple sequence repeat (SSR) types detected in eight chloroplast genomes; (**D**) total numbers of different SSR motifs in eight chloroplast genomes.

Repeat analyses of three newly sequenced cp genomes showed 13/13/13 (*G. siphonantha*/*G. officinalis*/*G. dahurica*) palindromic repeats, 12/11/11 dispersed repeats, and 7/6/6 tandem repeats (Figure 3A,B) with the repeat length range from 15 to 38 bp (Tables S1 and S2). The numbers and distribution of all repeat types were similar and conserved in these three cp genomes. Overall, 32/30/30 repeats were detected in three cp genomes. Similarly, 37, 34, 34, and 37 repeats were found in previously reported *G. crassicaulis*, *G. robusta*, *G. straminea*, and *G. tibetica* cp genomes (Figure 3A,B). Unexpectedly, 61 repeats, including 28 dispersed repeats, 18 palindromic repeats and 15 tandem repeats, were found in the cp genome of *G. macrophylla*. We found most of repeats in eight cp genomes were located in the intergenic or intron regions, and only a few repeats were distributed in protein-coding regions (*ycf1*, *ycf2*, and *psaA*) (Tables S1 and S2). Simple sequence repeats (SSRs) consisting of 1–6 bp repeat unit are distributed throughout the genome. In our study, perfect SSRs in eight *Gentiana* cp genomes were detected. The results showed that Mono-nucleotide repeats were most abundant type, followed by Tetra-nucleotides, Di-nucleotides and Tri-nucleotides. The penta- and hexa-nucleotides were very rare across the cp genomes (Figure 3C,D). Most SSRs are located in intergenic regions, but some were found in *rpoC2*, *rpoC1*, *atpB*, *ndhF*, and *ycf1* coding genes (Table S3). To investigate the evolutionary characteristics of cpDNA genes in eight *Gentiana* cp genomes and estimate selection pressures, nonsynonymous (dN), synonymous substitution rates (dS), and the ratio of dN/dS were calculated for 78 protein-coding genes (Table S4). We obtained 771 pairwise comparison results of dN/dS values and the remaining could not be calculated due to dS = 0. Only four genes (*accD*, *clpP*, *matK*, and *ycf1*) had dN/dS values ≥1 indicating that they had undergone positive selection.

**Figure 4:**
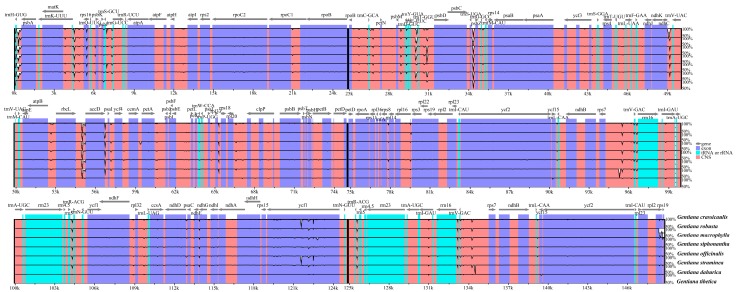
mVISTA percent identity plot comparing the eight chloroplast genomes of *Gentiana* sect. *Cruciata* with *G. crassicaulis* as a reference. The *y*-axis represents the percent identity within 50–100%. Genome regions are color-coded as protein coding (purple), rRNA, or tRNA coding genes (blue), and noncoding sequences (pink).

**Figure 5:**
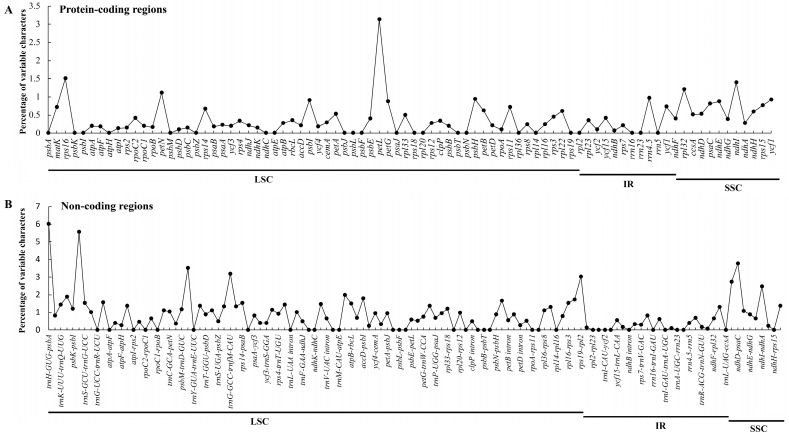
Percentage of variable characters in eight aligned chloroplast genomes of *Gentiana* sect. *Cruciata*. (**A**) Coding region; (**B**) Noncoding region.

To understand the level of sequence divergence, comparative analysis among eight *Gentiana* cp genomes was performed using mVISTA with the annotation of *G. crassicaulis* as a reference. The cp genomes within sect. *Cruciata* showed high sequence similarities with identities of only a few regions below 90%, indicating a high conservatism of these chloroplast genomes (Figure 4). The single-copy regions and intergenic regions were more divergent than the IR regions and genic regions (Figure 5). According to the comparative analyses, some hotspot regions for genome divergence that could be utilized as potential genetic markers to elucidate the phylogenies and to discriminate the species in sect. *Cruciata*. These regions were *psbA-trnH*, *trnK-rps16*, *rps16-trnQ*, *trnS-trnG*, *trnE-trnT*, *psbM-trnD*, *trnT-psbD*, *trnS-psbZ*, *ndhC-trnV*, *atpB-rbcL*, *rbcL-accD*, *accD-psbI*, *rpl33-rps18*, *trnR-trnA*, and *trnV-rps7* (Figure 4).

### 2.3. Development of InDel Markers to Discriminate Species of Sect. Cruciata

**Figure 6:**
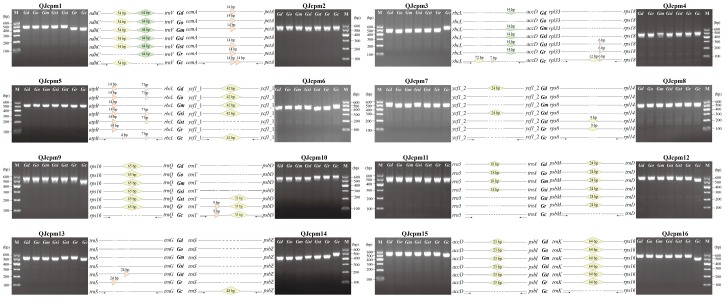
Validation of 16 molecular markers derived from InDel regions of eight chloroplast genomes of *Gentiana* sect. *Cruciata*. Inserted sequences and tandem repeats are designated by diamonds and triangle, respectively. Solid and dotted lines indicate conserved and deleted sequences, respectively. Left and right black arrows indicate forward and reverse primers, respectively. Abbreviated species names were shown on schematic diagrams: *Gd*, *G. dahurica*; *Go*, *G. officinalis*; *Gm*, *G. macrophylla*; *Gsi*, *G. siphonantha*; *Gst*, *G. straminea*; *Gr*, *G. robusta*; *Gc*, *G. crassicaulis*; M, D600 DNA ladder.

Based on the alignment of complete cp genome sequences, the 16 most InDel-variable loci were selected as candidate DNA markers for authentication (Table S5). After PCR amplification, these 16 markers could successfully amplify the expected polymorphic band sizes (Figure 6). Some of these 16 markers had unique amplicon sizes specific to different *Gentiana* species (Figure 6). Especially five markers (QJcpm9, QJcpm12, QJcpm14, QJcpm15, and QJcpm16) were specific to *G. crassicaulis*, which all derived from long InDels in the intergenic regions including *rps16-trnQ*, *psbM-trnD*, *trnS-psbZ*, a*ccD-psbI*, and *trnK-rps16.* The marker QJcpm1 was specific to *G. robusta* and *G. crassicaulis* and was derived from a 54 and 64 bp InDel in the *ndhC-trnV* region. The QJcpm2 marker derived from 14 bp tandem repeat (TR) in *cemA-petA* region was specific to *G. siphonantha* and *G. crassicaulis*. QJcpm3 marker, which was specific to *G. officinalis* and *G. crassicaulis*, was derived from 72, 14 bp InDels, and 7 bp TR in *rbcL-accD* region. Three markers (QJcpm4, QJcpm10, and QJcpm11) were specific to *G. straminea*, *G. robusta*, and *G. crassicaulis*. QJcpm4 marker was derived from 12 bp InDels and 6 bp TR in the *rpl33-rps18* region; QJcpm10 marker was derived from 9 bp TR and 33 bp InDel in the *trnT-psbD*; QJcpm11 marker was derived from 18 bp InDel in *rrn5-trnA* region. The QJcpm5 marker, which was derived from 14, 4, and 7 bp TR in *atpB-rbcL*, was specific to *G. macrophylla*, *G. robust*a, and *G. crassicaulis*. Three markers QJcpm6, QJcpm8, and QJcpm13 were derived from a 42 bp InDel in *ycf1*, 9 bp InDel in *rps8-rpl14* region, and 24 bp TR in the *trnS-trnG* region, respectively, and were specific to *G. straminea* and *G. robusta*. The marker QJcpm7, which was specific *G. dahurica* and *G. siphonantha*, was also derived from 24 InDel in *ycf1* CDS region. Our validation results indicated all these markers can be used to identify species in sect. *Cruciata*. 

### 2.4. Phylogenetic Relationships of Species Belong to Sect. Cruciata

**Figure 7:**
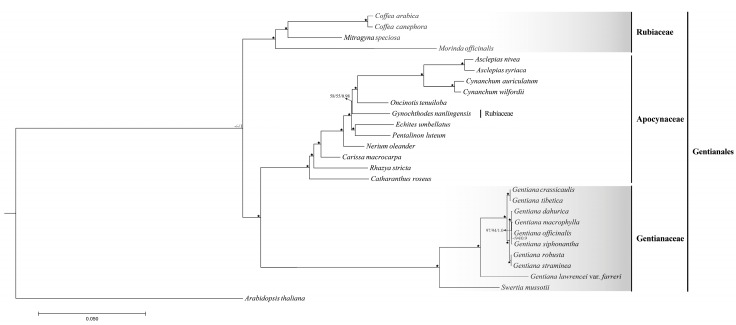
Phylogenetic relationships of species belong to *Gentiana* sect. *Cruciata* inferred from MP/ML/BI analysis based on complete chloroplast genome sequences. The numbers associated with each node are bootstrap support and posterior probability values, and the symbol 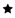 in the phylogenetic tree indicated that the support value of branch is 100/100/1.0.

Here, 27 cp genomes were retrieved to infer the interspecific relationships of eight species in sect. *Cruciata* as well as to clarify the phylogenetic relationships of some Gentianales species (Table S6). Phylogenetic analyses were performed using Maximum parsimony (MP), Maximum likelihood (ML) and Bayesian inference (BI) methods, and *Arabidopsis thaliana* was set as outgroup. Three different datasets including complete cp genomes, 70 shared protein-coding genes (PCGs) and the most conserved regions (TMCRs) of cp genomes were used to construct the phylogenetic trees. The results showed the same phylogenetic signals for these three datasets and the phylogenetic trees inferred from MP/ML/BI methods also shared identical topologies (Figure 7, Figures S2 and S3). In these phylogenetic trees, we found all the species of sect. *Cruciata* formed a monophyletic clade a with high bootstrap and BI support values and clustered with another two Gentianaceae species (*G. lawrencei* and *Swertia mussotii*) in the same clade [29,30]. Of these species, *G. macrophylla*, *G. officinalis*, and *G. siphonantha* showed paraphyletic relationships with each other and formed a monophyletic clade with *G. dahurica*. *G. tibetica* and *G. crassicaulis* formed a monophyletic clade and located in the basal position of these eight species in sect. *Cruciata.* Interestingly, *G. robusta* and *G. straminea* with similar morphological characteristics were clustered in a monophyletic clade with a high resolution value. In addition, our phylogenetic results supported the monophyly of two families, including Apocynaceae and Rubiaceae, in the order Gentianales. Unexpectedly, *Gynochthodes nanlingensis* (*Morinda nanlingensis*) belongs to Rubiaceae was embed in the Apocynaceae species.

## 3. Discussion

Three cp genomes of sect. *Cruciata* were sequenced using Illumina Hiseq platform, which provided more resources for evolutionary and genetics studies of *Gentiana*. The cp genomic information presented in this study will also contribute to the conservation and management of wild resources of sect. *Cruciata*. Although a recent research reported that 11 ndh genes had been lost in the cp genomes of *Gentiana* sect. *Kudoa* [31], eight cp genomes of sect. *Cruciata* analyzed in present study are rather conserved in gene structures, contents and arrangement, and no significant structural rearrangements, such as inversions or gene relocations, were detected. Of these eight species, *G. macrophylla* has the largest cp genome size and other species showed minor differences in genome size. The length variations of these cp genomes may result from the length of intergenic regions, similar result has been reported for *Paris* (Melanthiaceae) cp genomes [18]. 

All the eight cp genomes of sect. *Cruciata* had the same protein-coding genes, tRNA and rRNA genes. We found that exon2 of *rps16* gene was lost in three newly sequenced cp genomes, and *rps16* in other cp genomes also showed same structure. Therefore, *rps16* pseudogene may commonly exist in the genus *Gentiana* [26]. And *infA* gene, which contains internal stop codons, was also inferred as pseudogene in these species. This pseudogene had been reported in many species [32,33,34,35]. Except for cp genome of *G. macrophylla*, the remained cp genomes showed minor variations in the junctions between the SSC and IRs regions. As most species of sect. *Cruciata* derived from a common radiation and usually showed closely interspecific relationships, we thus speculated that highly conserved nature of cp genomes resulted in the similar gene distributions at SC/IR boundaries. 

Repeat structure plays an important role in genomic rearrangement, recombination, and sequence divergence in plastomes [36,37,38]. In the present study, cp genome of *G. macrophylla* has the largest number of repeats, while the number of repeats was similar in other cp genomes. Most of the repeated regions in different species showed similar characteristics and most repeats were located in intergenic regions or in *ycf1*/*pasA*. Repeats in these genes are commonly observed in other angiosperm lineages [22,32,39]. Cp microsatellites (cpSSR) usually showed high polymorphism within the same species and which are potentially useful markers for population genetics [40]. Here, 326 SSRs varying in number and type between eight major *Gentiana* species, and the most abundant repeat type was found to be stretches of mononucleotides (A/T). Similar to the distribution status of dispersed and tandem repeats, most cpSSRs were observed in noncoding regions, and only small proportion were found in coding regions. CpSSRs located in noncoding regions of the cp genome are generally short mononucleotide tandem repeats and commonly showed intraspecific variation in repeat number [15]. Therefore, cpSSRs derived from eight *Gentiana* species in this study are expected to be useful for the genetic diversity studies in *Gentiana*. As the wild resources of some *Gentiana* species were dramatically declined due to overexploitation, we thought these species need to transplant or cultivate in order to preserve their germplasm resources. We believe the obtained SSRs among these chloroplast genomes will also be useful for the domestication and breeding of *Gentiana* species.

Sequence divergence of the coding genes was observed between different species. Our analyses indicated that all of cp genes showed a low sequence divergence (dS < 0.1) and most cp genes were under purifying selection (dN/dS < 1); similar results were reported for other cp genomes [32,41,42]. Only four genes (*accD*, *clpP*, *matK*, and *ycf1*) were under positive selection. Previous research reported that *accD* and *clpP* genes had a high evolution rate in *Fagopyrum* species [43,44], we thus presumed that these genes may have a high evolution rate in *Gentiana* species. One other gene (*matK*) was highly divergent in Caryophyllaceae, and comparative cp genomes analyses of Myrtaceae also indicated *matK* was under positive pressure [45,46]. The *ycf1* gene with unknown functions showed a biased higher value for dN/dS ratio compared to dS value indicating that this gene evolved at a faster rate. It has also been shown to be subject to positive selection in many angiosperms [20,22,32,44,45].

DNA barcodes are defined as the short DNA sequences with a sufficiently high mutation rate to discriminate a species within a given taxonomic group and are confirmed as reliable tools for the identification of plant species [16,47]. Previously, *rbcL*, *trnH-psbA*, and *matK* were considered as “core” plant barcodes for species identification, but they often have limited resolutions at species level [18]. Previous research showed that three commonly used barcodes in some cases may not be suitable to authenticate the medicinal plant in section *Cruciata* [24,25]. Therefore, seeking for more effective DNA barcodes with high evolutionary rates is very important for the molecular identification of species in *Gentiana* sect. *Cruciata*. The complete cp genome has a conserved sequence from 110k to 160k bp, which far exceeds the length of commonly used molecular markers and provides more variation to distinguish closely related species [12,16]. Therefore, some mutation hotspot regions, including *trnK-rps16*, *rps16-trnQ*, *trnS-trnG*, *trnE-trnT*, *trnT-psbD*, *trnS-psbZ*, *ndhC-trnV*, *rbcL-accD*, *accD-psbI*, *trnR-trnA*, *trnV-rps7*, and *ycf1*, detected from the cp genomes can provide more specific DNA barcodes for the authentication of medicinal materials of sect. *Cruciata* and also provide sufficient genetic markers for resolving the phylogeny of Gentianaceae.

We developed the specific markers for species authentication of sect. *Cruciata* based on the hotspot regions derived from cp genomes. Most of these markers were derived from the intergenic regions of cp genomes and showed high interspecific polymorphism. Previous molecular identification of *Panax*, *Zanthoxylum*, and *Eclipta* species also indicated that chloroplast-derived genetic markers had high discriminatory powers [12,14,48]. Therefore, specific markers developed from the comparative cp genomes were superior than the commonly used markers for identifying the closely related species. Especially for medicinal plants, these specific genetic markers are more effective in the authentication of their source plants. We found two InDels (42 and 24 bp) in the *ycf1* gene, which can be used to distinguish species in sect. *Cruciata*. *Ycf1*, which encodes a protein of approximately 1800 amino acids with unknown function, is the second largest gene in the cp genome. Because the sequence of *ycf1* is too long and too variable for designing universal primers, it has received little attention for DNA barcodes at low taxonomy [18,49]. But two markers derived from *ycf1* gene showed high PCR efficiency and polymorphism in species of sect. *Cruciata*, and could be used as specific barcodes for the authentication of *Gentiana* species. Although our study provided 16 genetic markers which had enough interspecies polymorphism for species identification, some of the markers were usually specific to two species. We thus suggest a combination of several markers should be considered for credible authentication between different species in genus *Gentiana*.

We inferred the phylogenetic relationships of sect. *Cruciata* using complete cp genomes. Three different methods (MP/ML/BI) were used to rebuilt the phylogenetic trees based on different datasets (cp genomes, 70 shared PCGs, and TMCRs), and the derived phylogenetic trees shared identical topology. All the species of sect. *Cruciata* formed a monophyletic clade with high bootstrap and BI support values. This result is comparable with the previous phylogenetic research based on four cpDNA fragments [1]. Four species, including *G. dahurica*, *G. macrophylla*, *G. siphonantha*, and *G. officinalis*, were clustered in the same clade with high support values. Although the flower color of *G. officinalis* was different from other three species, it shared similar morphological and chemical characters with *G. macrophylla* [50]. We found that *G. straminea* was closely related to *G. robusta*. *G. robusta* may have originated from introgression between *G. straminea* and another relative species, and these two species are usually closer to each other [26,51]. Two species, *G. tibetica* and *G. crassicaulis* were clustered in the same clade and located in the basal position in the clade of sect. *Cruciata*. However, a previous phylogenetic result indicated that *G. tibetica* was closely related to *G. straminea* and *G. robusta* [1]. As *G. tibetica* and *G. crassicaulis* distributed sympatrically in Tibet and intermediate types were produced by introgression between these two species [52], we thus inferred these two species should be closely related. In addition, based on the phylogenetic results, we found that the family Gentianaceae was closer to family Apocynaceae than to family Rubiaceae in order Gentianales. Previous phylogenetic studies of order Gentianales resulted in similar findings, but with relatively low support values [53,54]. Although our result confirmed the monophyly of section *Cruciata* and primarily elucidated the phylogeny of Gentianales based on available cp genomes, more complete cp genome sequences are needed to resolve the comprehensive phylogenies of this section, especially since limited taxon sampling may produce discrepancies in tree topologies [15,55].

## 4. Materials and Methods

### 4.1. Plant Materials and DNA Isolation

Samples of *G. dahurica*, *G. siphonantha* and *G. officinalis* were collected from Tianzhu (102.54° E, 37.01° N), Sunan (98.05° E, 39.55° N) and Yuzhong (104.05° E, 35.78° N) Counties in Gansu Province, China. Young leaves of three species were collected and immediately dried with silica gel for further DNA isolation. Total genomic DNA was isolated from each sample using the modified Cetyl Trimethyl Ammonium Bromide (CTAB) method [56]. The quantity and quality of extracted genomic DNA was determined by gel electrophoresis and NanoDrop 2000 Spectrophotometer (Thermo Scientific, Carlsbad, CA, USA).

### 4.2. Chloroplast Genome Sequencing, Assembly and Annotation

The DNA Library with insert size of 200 bp was prepared according to the description by Zhou et al. [32], and sequenced using Illumina Hiseq^TM^ 2500 platform (Illumina Inc., San Diego, CA, USA) with the average read length of 125 bp. The obtained raw reads were filtered with the NGS QC Toolkit_v2.3.3 (National Institute of Plant Genome Research, New Delhi, India) [57]. Adapter sequences and low-quality reads with Q-value ≤ 20 were removed. Filtered paired-end reads were firstly mapped to the chloroplast genome of *Gentiana straminea* (KJ657732) by using the Bowtie 2-2.2.6 (University of Maryland, College Park, MD, USA.) with default parameter [58]. And then the matched paired-end reads were de novo assembled using SPAdes-3.6.0 (St. Petersburg Academic University, St. Petersburg, Russia) [59]. After de novo assembly, the resultant scaffolds were further assembled using a baiting and iteration method based on Perl script MITObim_1.9.pl (University of Oslo, Oslo, Norway) [60]. Finally, all obtained reads were mapped to the spliced cp genome sequence using Geneious 10.1 (Biomatters Ltd., Auckland, New Zealand) in order to avoid assembly errors. The four junction regions between the IRs and SSC/LSC were confirmed by PCR amplification and Sanger sequencing (Primers and sequencing results are listed in Table S7). The cp genome genes were annotated with the online program Organellar Genome Annotator (DOGMA) [61], and the primary annotated results were manually verified according to the annotation information from other closely related species. The circular plastid genome maps were drawn using the online program OrganellarGenome DRAW (Max planck Institute of Molecular Plant Physiology, Potsdam, Germany) [62] and three newly sequenced cp genome were deposited in GenBank (MH261259–MH261261). 

### 4.3. Repeat Structure, Genome Comparison and Sequence Divergence

Dispersed and palindromic repeats within the cp genomes were identified using REPuter (University of Bielefeld, Bielefeld, Germany) with a minimum repeat size of 30 bp and a sequence identity > 90% [63]. Tandem repeat sequences were searched using the Tandem Repeats Finder program (Mount Sinai School of Medicine, New York, NY, USA) with the following parameters: 2 for alignment parameters match, 7 for mismatch and InDel, respectively [64]. Simple sequence repeats (SSRs) were predicted using MISA perl script (Institute of Plant Genetics and Crop Plant Research, Gatersleben, Germany) with the parameters of ten for mono, five for di-, four for tri-, and three for tetra-, penta, and hexa-nucleotide motifs [65]. The nonsynonymous (dN), synonymous (dS), and dN/dS values of each protein coding gene were calculated using PAML packages 4.0 (University College London, London, UK) with Yang and Nielsen (YN) algorithm to detect whether selective pressure exists for plastid genes [66]. The cp genome gene distribution of eight *Gentiana* species was compared and visualized using mVISTA software with the annotation of *G. crassicaulis* as a reference [67]. To examine mutation hotspot regions of the cp genomes of eight *Gentiana* species, the percentages of variable characters for each coding and noncoding regions were analyzed using the method described by Zhang et al. [68].

### 4.4. Development and Validation of the InDel Molecular Marker

In order to validate interspecies polymorphisms within the chloroplast genomes and develop DNA genetic markers for identifying species belong to sect. *Cruciata*, specific primers were designed using Primer 3 based on the mutational hotspot regions found in these *Gentiana* chloroplast genomes [69]. PCR amplifications were performed in a reaction volume of 25 μL with 12.5 μL 2× Taq PCR Master Mix, 0.4 μM of each primer, 2 μL template DNA and 10.1 μL ddH_2_O. All amplifications were carried out in SimpliAmp™ Thermal Cycler (Applied Biosystems, Carlsbad, CA, USA) as follow: denaturation at 94 °C for 5 min, followed by 30 cycles of 94 °C for 50 s, at specific annealing temperature (Tm) for 40 s, 72 °C for 90 s and 72 °C for 7 min as final extension. PCR products were visualized on 2% agarose gels after staining with ethidium bromide and then the DNA fragments were sequenced by Sangon Biotech (Shanghai, China) (Sequencing results are listed in Table S8).

### 4.5. Phylogenetic Analysis

The complete chloroplast genomes of 26 Gentianales species were recovered to clarify the phylogenetic relationships of sect. *Cruciata* and the cp genome of *Arabidopsis thaliana* was set as outgroup. In order to obtain a reliable result, phylogenetic analyses were implemented based on different cp genome datasets. On the one hand, whole cp genome sequences and 70 common cp protein-coding genes (PCGs) were separately used to infer the phylogenetic relationships of these species. On the other hand, multi-gene alignment matrix, which contained the most conserved regions (TMCRs) of cp genome was generated using HomBlocks (Ocean University of China, Qingdao, China) [70], was used to understand the phylogenetic relationships at cp genome level. Alignments were constructed using MAFFT v7.308 (Osaka University, Suita, Japan) with default parameters and the best-fit nucleotide substitution model (General Time Reversible + Invariant + Gamma, GTR + I + G) was determined with Modeltest 3.7 (Brigham Young University, Provo, UT, USA) [71,72]. Maximum parsimony (MP) analyses of the resulting alignments from different datasets were performed using PAUP 4.0b10 (Smithsonian Institution, Washington, DC, USA) [73]. Maximum likelihood (ML) analyses were performed using RAxML 8.1.24 (Heidelberg Institute for Theoretical Studies, Heidelberg, Germany) with GTR + I + G nucleotide substitution model [74]. The reliability of each tree node was tested by bootstrap analysis with 1000 replicates. Bayesian analyses were also conducted with MrBayes v3.2.6 (Swedish Museum of Natural History, Stockholm, Sweden) [75] under the same substitution model (GTR + I + G). The Markov chain Monte Carlo (MCMC) algorithm was run for one million generations, with one tree sampled every 100 generations. The first 25% of trees were discarded as burn-in to construct majority-rule consensus tree and estimate posterior probabilities (PP) for each node.

## Figures and Tables

**Table 1:** Summary of complete chloroplast genomes for eight *Gentiana* species.

**Name of Taxon**	***G. dahurica***	***G. siphonantha***	***G. officinalis***	***G. straminea***
Genome length	148,803	148,908	148,879	148,991
LSC length	81,154	81,121	81,119	81,240
SSC length	17,093	17,113	17,088	17,085
IR length	25,278	25,337	25,336	25,333
Total gene number	112	112	112	112
No. of protein coding genes	78	78	78	78
No. of tRNA genes	30	30	30	30
No. of rRNA genes	4	4	4	4
GC content in genome (%)	37.7	37.7	37.7	37.7
**Name of Taxon**	***G. crassicaulis***	***G. robusta***	***G. tibetica***	***G. macrophylla***
Genome length	148,776	148,911	148,765	149,916
LSC length	81,164	81,164	81,163	82,911
SSC length	17,071	17,085	17,070	17,095
IR length	25,271	25,333	25,266	24,955
Total gene number	112	112	112	112
No. of protein coding genes	78	78	78	78
No. of tRNA genes	30	30	30	30
No. of rRNA genes	4	4	4	4
GC content in genome (%)	37.7	37.7	37.7	37.7

## Supplementary Materials

Supplementary materials can be found at http://www.mdpi.com/1422-0067/19/7/1962/s1.
