# Supplementary material for: Comparative Chloroplast Genome Analyses of Species in Gentiana section Cruciata (Gentianaceae) and the Development of Authentication Markers
Source: Int J Mol Sci. 2018 Jul 5;19(7):1962. doi: 10.3390/ijms19071962 (PMC6073106; doi:10.3390/ijms19071962)
Supplement: Supplementary file 1 [file ijms-19-01962-s001.zip › Supplementary Materials/ijms-311023-suppl-fig.pdf]

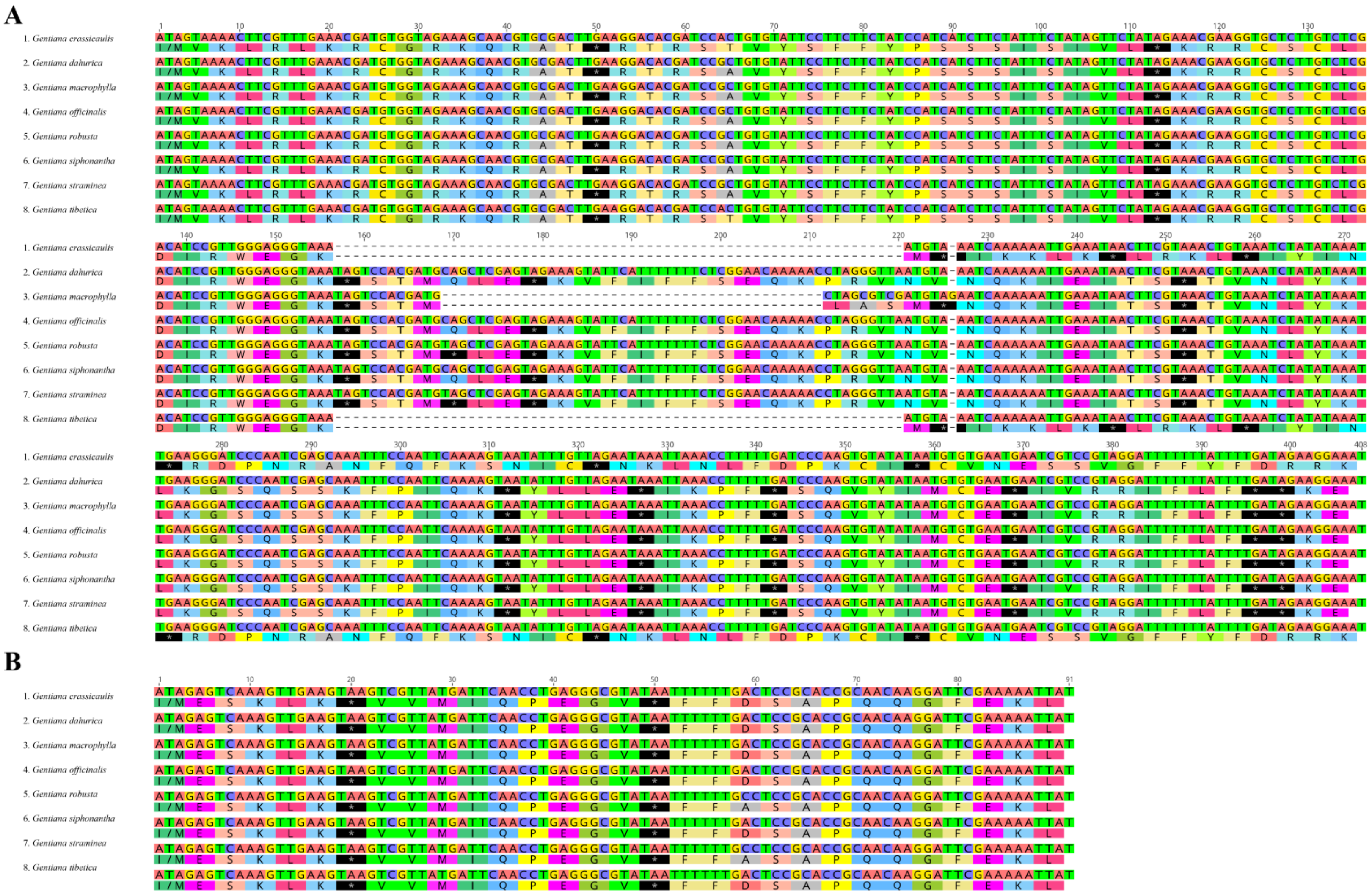

Figure S1. Alignment of two pseudogenes in the eight *Gentiana* species chloroplast genome. (**A**) *rps16*. (**B**) *infA*. The black box with an asterisk represents stop codons.

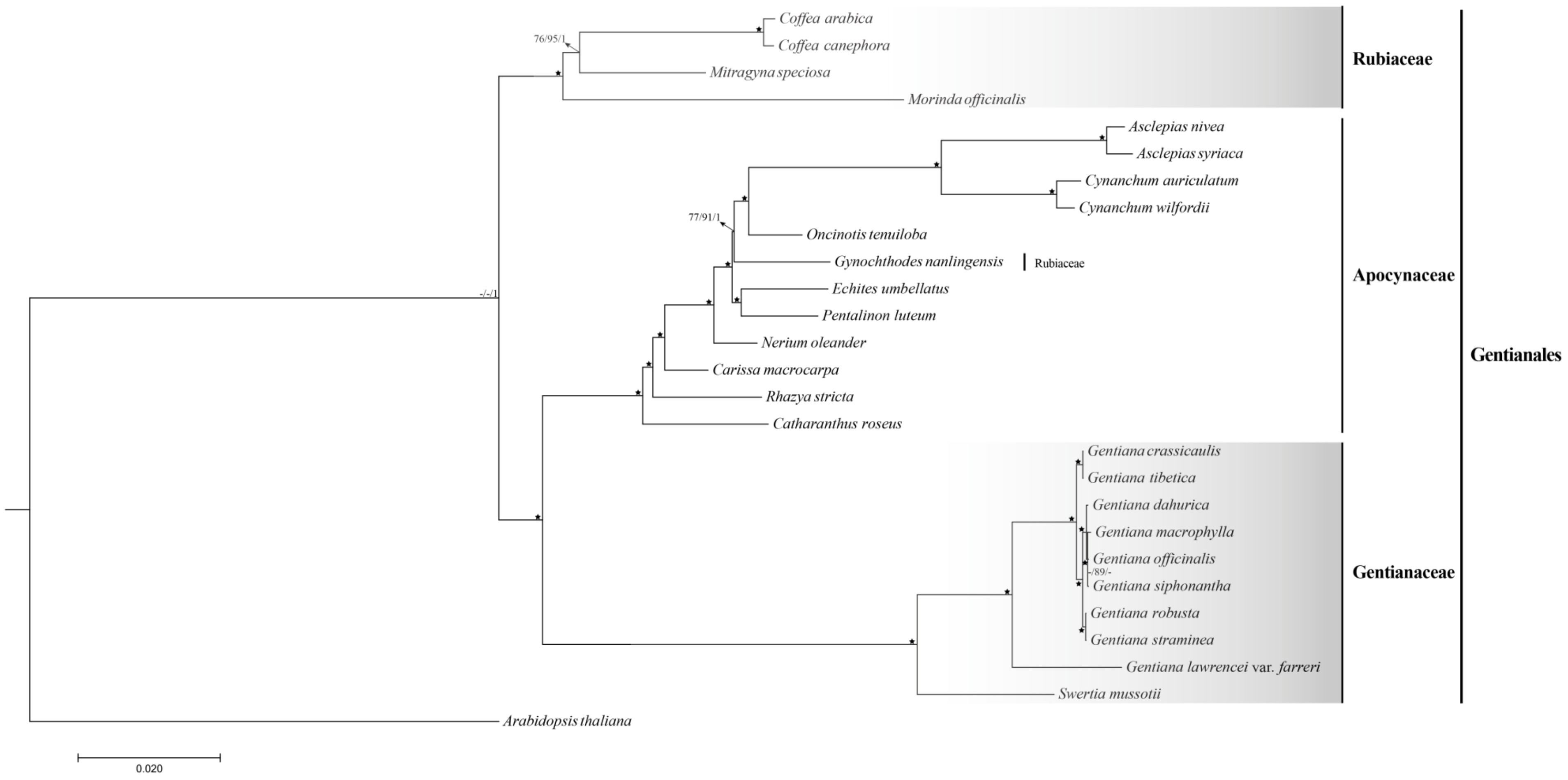

Figure S2. Phylogenetic relationships of species belong to *Gentiana* sect. *Cruciata* inferred from MP/ML/BI analysis based on 70 shared PCGs. The numbers associated with each node are bootstrap support and posterior probability values, and the symbol ★ in the phylogenetic tree indicated that the support value of branch is 100/100/1.0.

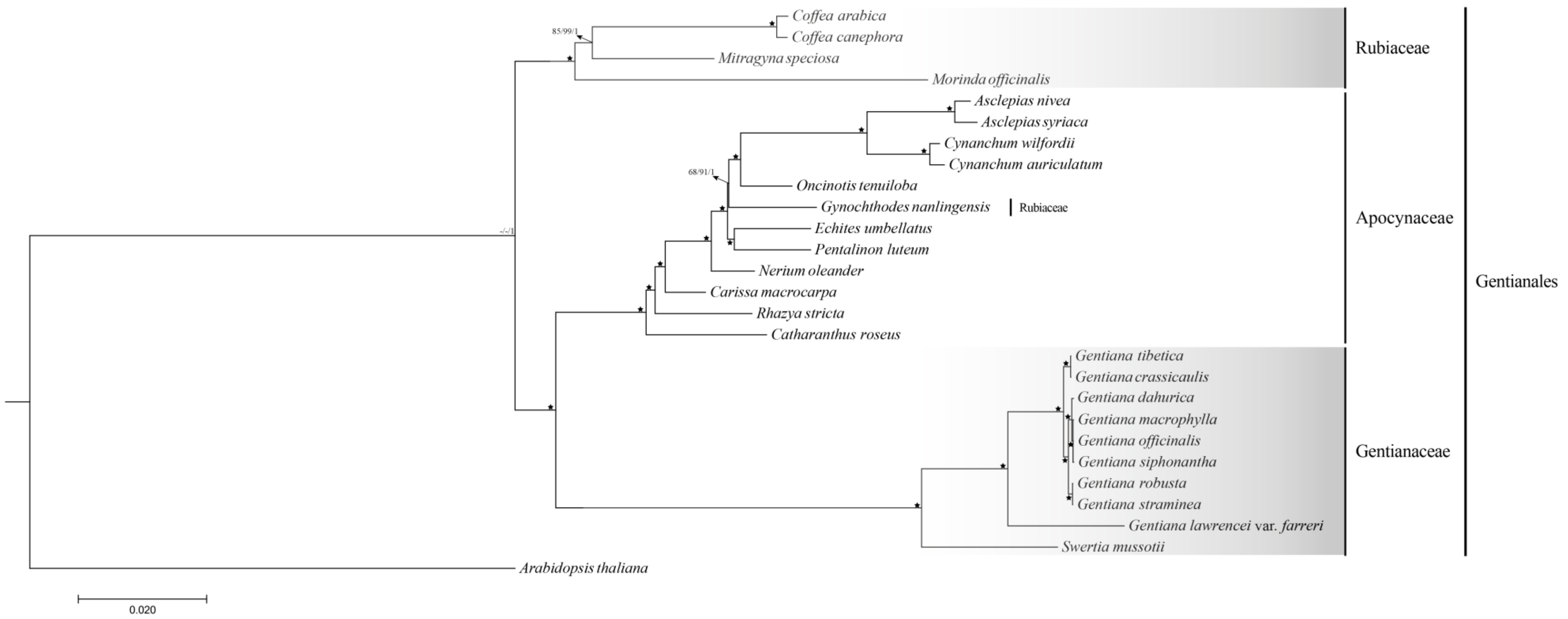

Figure S3. Phylogenetic relationships of species belong to *Gentiana* sect. *Cruciata* inferred from MP/ML/BI analysis based on TMCRs of chloroplast genomes. The numbers associated with each node are bootstrap support and posterior probability values, and the symbol ★ in the phylogenetic tree indicated that the support value of branch is 100/100/1.0.
